# Evolution of *M. bovis* BCG Vaccine: Is Niacin Production Still a Valid Biomarker?

**DOI:** 10.1155/2015/957519

**Published:** 2015-01-28

**Authors:** Sarman Singh, Manoj Kumar, Pragati Singh

**Affiliations:** ^1^Division of Clinical Microbiology & Molecular Medicine, Department of Laboratory Medicine, All India Institute of Medical Sciences, New Delhi 110029, India; ^2^National Polio Surveillance Project, Country Office for India, World Health Organization, Mathura 281001, India

## Abstract

BCG vaccine is usually considered to be safe though rarely serious complications have also been reported, often incriminating contamination of the seed strain with pathogenic *Mycobacterium tuberculosis*. In such circumstances, it becomes prudent to rule out the contamination of the vaccine seed. *M. bovis* BCG can be confirmed by the absence of nitrate reductase, negative niacin test, and resistance to pyrazinamide and cycloserine. Recently in India, some stocks were found to be niacin positive which led to a national controversy and closer of a vaccine production plant. This prompted us to write this review and the comparative biochemical and genotypic studies were carried out on the these contentious vaccine stocks at the Indian vaccine plant and other seeds and it was found that some BCG vaccine strains and even some strains of *M. bovis* with eugenic-growth characteristics mainly old laboratory strains may give a positive niacin reaction. Most probably, the repeated subcultures lead to undefined changes at the genetic level in these seed strains. These changing biological characteristics envisage reevaluation of biochemical characters of existing BCG vaccine seeds and framing of newer guidelines for manufacturing, production, safety, and effectiveness of BCG vaccine.

## 1. Introduction

BCG, an attenuated strain of* Mycobacterium bovis* (*M. bovis*), has been used in more than 182 countries or territories as a prophylactic vaccine against tuberculosis (TB), for more than 90 years, albeit amidst a considerable controversy related to its efficacy. The true efficacy of BCG has been difficult to understand due to many experimental variables [1].* M. bovis* is the etiological agent of bovine tuberculosis and is closely related to* Mycobacterium tuberculosis *(*M. tuberculosis*) in the* M. tuberculosis* complex (MTBC), which consists of* M. tuberculosis, M. bovis, M. bovis *BCG,* M. africanum, M. canettii, M. microti, M. caprae, *and* M. pinnipedii. *The* M. bovis* mainly infects cattle (*Bos taurus*), but it can infect other mammalians including humans [2, 3]. The BCG vaccine undoubtedly provides protection against childhood disseminated form of TB including TB meningitis. However its efficacy against pulmonary TB in adults has been reported to give variable results [4]. In 2011, World Health Organization (WHO) monitored study revealed that protection levels ranged from 53% in Equatorial Guinea and 54% in Ethiopia to more than 99.5% in India and China [5]. Its efficacy in programmed mode is reported to be more than 80% [6]. So far more than 3 billion doses of BCG vaccine have been given since 1948, and by and large it is considered safe [7]. However localized abscess formation, disseminated disease, and regional lymphadenopathy, especially in immunocompromised hosts are rare but well-recognized complications [8].

An estimated 8.6 million new cases and 1.3 million deaths due to tuberculosis occur every year [9]. Almost all cases of tuberculosis are caused by* M. tuberculosis*, and share of* M. bovis* is less than 1.4 percent of all pulmonary tuberculosis cases outside of Africa. Though, in Africa,* M. bovis* accounts for approximately 2.8 percent of cases of pulmonary tuberculosis, for a crude incidence of 7 cases per 100,000 populations [10], the global proportion of* M. bovis* is higher among patients with extrapulmonary tuberculosis, since the pathogen is frequently acquired via oral ingestion and gastrointestinal disease is an important clinical manifestation [11].

## 2. Historical Aspect of BCG

The original BCG vaccine “strain” was derived from an isolate of* M. bovis*. Since 1900, Albert Calmette (1863–1933) began his research on the* M. bovis *strain, which had been isolated from the milk of an infected cow by veterinarian Jean-Marie Camille Guérin (1872–1961) in 1904. In addition, the “strain” was named Bacillus of Calmette and Guérin. They cultivated these bacilli in a medium containing glycerin and potato, but they found that there was difficulty in the production of homogenous suspension of the bacilli. To make the bacteria homogenous they added ox bile to the medium and to their revelation, they found that the additive has lowered the virulence of bacteria. This unexpected observation became source of vaccine production from the attenuated tubercle bacilli [12]. Benjamin Weill-Hall (1875–1958), a French pediatrician and bacteriologist, was the first to feed the vaccine to infants in Paris who were at a risk for the disease. However, in 1908, Camille Guérin and Benjamin Weill-Hall, both at the Institute Pasteur in Lille, France, began attenuating the* M. bovis *by passing it through a growth medium they had developed specifically for this purpose and an actual BCG vaccine was thus developed at the Pasteur Institute in Lille and was first given to humans in 1921. The first formal trial of BCG outside France was organized among the North American Indians in the 1930s [13]. By the late 1940s, several studies provided evidence favouring its utility in protection against tuberculosis. For this, the original culture was subcultured and distributed to several laboratories throughout the world, where the vaccine strain was called BCG and was maintained by continuous subcultures. After many years, the various strains maintained in different laboratories were found to be no longer identical to each other. In fact, it was likely that various strains maintained by continuous subculture continued to undergo undefined genetic changes. Indeed, even the “original” strain of BCG maintained at Paris also continued to change its characteristic during the subcultures. To limit the genetic changes the procedures needed to maintain the strain were modified time to time. Currently, the* M. bovis* BCG is maintained by using a “seed-lot” production technique to limit further genetic variations.

Presently, five main strains or seed-lots, accounting for more than 90% of the vaccine produced, are used worldwide with each strain possessing different biological characteristics. These strains are Pasteur 1173 P2, the DANISH 1331, the Glaxo 1077 (derived from the DANISH strain), the Russian BCG-I, the Tokyo 172-1, and the Moreau RDJ strains [24]. Confusions are generated by the vague terminologies used by individual stakeholders (e.g. “American” strain), varying nomenclature (e.g., BCG Brazil is the synonym of BCG Moreau, although Moreau was from Uruguay), and unusual corporate events (e.g., Pasteur-MeÂrieux-Connaught produces BCG- Glaxo except in Canada where BCG-Connaught is used) [25]. Articles on BCG molecular biology reflect this confusion, with studies employing different strains, attributed to different historical periods [26]. In the extent of different vaccine efficacy and safety in humans, it is not clear at present; but some differences in the molecular and genetic characteristics are known and each BCG has been called by the location where it is produced; for example, BCG (Paris), BCG (Copenhagen), BCG (Tice), and BCG (Montreal).

In India, the BCG vaccination programme was started in 1948 and BCG vaccine laboratory was established in Madanpalle (Tamil Nadu, India). By 1960, the first round of mass BCG vaccination was completed in all states with about 254 million persons having been vaccinated by 1979. Yet BCG is one of the most controversial vaccines till today [27]. Since the 1950s, the reason for the failure of BCG in some populations has been a subject of debate, and to explain the observed variation, different hypotheses have been suggested [28]. The differences in the strain of BCG, the age at vaccination, or methodological differences are important factors [29]. One exception from this general rule is the consistent high efficacy when BCG is used to vaccinate newborns. Neonatal vaccination with BCG reports protection against the childhood manifestations of TB, especially the meningitis [30], but the efficacy decreases over a period of times, and therefore in the adult population the third world vaccine does not prevent against the later breakdown with pulmonary TB [28].

## 3. Biochemical and Genotypic Characteristics of BCG

Phenotypic characteristics have been a contentious issue and some strains are considered inferior over the others. Not only allegations of contamination with* M. tuberculosis* have been made occasionally, but also recently one batch of the Indian BCG vaccine was found to give niacin positive reaction and this led to the closure of vaccine plant in India. A high-level technical committee was formed by Government of India and one coauthor was part of this committee. As described in Table 1, the diagnostic features of BCG include growth in Lowenstein-Jensen and 7H11 media and in the modified Dubos liquid medium at 37°C; inhibition of growth in the presence of thiophene-2-carboxylic acid hydrazide; negative tests for niacin, catalase production at 68°C, nitrate reduction, Tween 80 hydrolysis; and a positive urease test [31]. On the basis of secreted proteins, MPB64 and MPB70 substrains of* M. bovis *BCG have been divided in two major groups: high and low producers of these proteins [16]. Polymerase chain reaction (PCR) and hybridization experiments indicate that the MPB64 gene is absent in the BCG substrains Pasteur, Glaxo, Copenhagen, and Tice. The species specificity of MPB64 and its occurrence in both* M. tuberculosis *and virulent strains of* M. bovis* may create further confusion [32]. Biochemical tests are currently used for the identification of bacterial species, including the genus* Mycobacterium* [33]. Several enzymes such as NAD and NADH quinone reductases, mycobacterial phospholipase A (MPLA) which catalyses the hydrolysis of lipids including Tween 80, and others appear to contribute to survival of the mycobacteria [34, 35]. An important virulence factor for* M. tuberculosis *and* M. bovis *is the nitrate reductase system. Chemically, BCG can be distinguished from* M. tuberculosis* by its weakly positive nitrate reduction ability. While the amidase test gives a strongly positive reaction to carbamide, whereas other amidases give negative results in Bônicke series [36].

Niacin production during the adaptation to hosts of several strains of biovars 1 to 4 can more readily switch on and switch off the genes. It is reported that* M. bovis *strains of the “European” type (which possess a single IS*6110 *fragment and which lack DR spacer sequences 39 to 43) branched off at an earlier stage than the other* M. bovis *strains. The* M. bovis *BCG has been reported as niacin test negative, nitrate reductase negative, and pyrazinamide and cycloserine resistance [37].

The elevated levels of nitrate reductase activity increase the virulence and consequently the success of some lineages of* M. tuberculosis *[38]. However, nitrite production has also been reported in some strains of* M. bovis *under different conditions such as longer incubation period and anaerobic conditions [39]. Both* M. tuberculosis *and* M. bovis *BCG express an anaerobic nitrate reductase (NarGHJI) activity and a* narG M. bovis *BCG mutant lacks the ability to reduce nitrate under anaerobic conditions [40]. A* narG* knockout mutant of BCG showed reduced virulence and reduced lung damage in severe combined immunodeficiency (SCID) mice. Thus* M. bovis *BCG, like* M. tuberculosis*, can form granulomas in different body sites and abscesses in various human tissues [41]. In MTB granuloma formation hypoxia plays an important role and this pathology is mediated by several enzymes including nitrate reductase [42, 43]. However, the role of hypoxia is not well defined in vaccine strain* M. bovis *BCG [44].

The human tubercle bacilli (*M. tuberculosis*) produce more niacin than other mycobacteria, and the detection of niacin production has been widely used for differentiating MTBC species from* M. bovis* which are usually niacin negative [45–47]. Recently, this biomarker created a huge crisis in Indian Government system, because the in-use lots of BCG vaccine were found to be niacin positive. Other manufacturers of the BCG vaccine alleged that the Indian seed-lot was contaminated with* M. tuberculosis*. Besides closing the vaccine production plant, the Government of India set up a technical committee to examine the controversy. Several seed-lots along with the alleged Indian lots were analyzed in Table 1. These results indicated that besides Indian seed-lot (BCG-P3) several other strains have also become niacin positive, without any evidence. The strain BCG-P3 has been found to lack genes normally present in* M. tuberculosis* but absent in BCG and was nonvirulent for Guinea pigs, ruling out contamination by* M. tuberculosis, *important fact. All vaccine producers are required to follow standard vaccine virulence testing guidelines as per WHO guidelines [45]. The literature also indicates that some bovine strains with eugenic-growth characteristic, mainly old laboratory strains, and some BCG vaccine strains may give a positive niacin reaction; on the other hand, certain* M. tuberculosis* strains with dysgenic-growth characteristics, such as isoniazid-resistant strains, may give a niacin negative reaction [48, 49].

## 4. *M. bovis* Genome and Biological Lifestyle

At genetic level also heterogeneity of niacin accumulation has been observed among BCG substrains. The* M. bovis *cell wall contains phenolic glycolipids that are absent in* M. tuberculosis*. A family of membrane-spanning proteins involved in the export of the phenolic cell wall glycolipids in the* M. bovis *genome (TbD1 locus) consists of the* mmp *genes [50]. A group of antigens of ESAT-6 family such as CPF-7 and CPF-10 which were originally described as T-cell antigens are secreted by* M. tuberculosis *[51], but these are also encoded by the genome of* M. bovis.* Other members of the family act in match-up; possibly in a mix-and-match array the interaction between ESAT-6 and CPF-10 is exhibited, whereas in* M. tuberculosis *the six members of the ESAT-6 familyare absent from the genome of* M. bovis *[52, 53].

## 5. *M. bovis* BCG Infection

BCG infections are infrequent, but rarely some children can develop localized or disseminated BCG infections. To differentiate these manifestations from other conditions recovery of the BCG strain of* M. bovis *from the pretentious focus is mandatory. The identification process of* M. bovis* is not simple as it relies on the isolation of the bacteria from the site of localized infection, usually the injection site, or from other tissues including the blood such as in case of disseminated infection. In adults, when BCG vaccine is used in bladder cancer therapy, dissemination can lead to fatal infection. Recently, molecular techniques have been frequently used to identify the true pathogens even when it is not culturable. The commonest molecular methods used to identify and confirm the diagnosis of BCG vaccine infections are PCR followed by single stranded conformation polymorphism (SSCP). The* pncA* gene is the most specific target due to the fact that polymorphic site at the 169 position of this gene,* M. bovis* BCG vaccine can be differentiated from* M. tuberculosis* using PCR-RFLP [54]. The standard mycobacterial culture techniques currently used in clinical microbiology laboratories are capable of identifying mycobacteria to the level of the* M. tuberculosis *complex. On the basis of morphology and biochemical criteria, it is difficult to differentiate between virulent* M. bovis *and* M. bovis *BCG. More sophisticated methods are probably needed to confirm a diagnosis of* M. bovis *BCG. Complications after BCG vaccination and the intrinsic resistance of* M. bovis *BCG to pyrazinamide, as well as knowledge on BCG infection, would be of particular interest to the clinician responsible for guiding therapy. After PCR-based diagnosis, therapy is based on drug susceptibility with BCG sensitive regimens, that is, isoniazid, rifampin, and ethambutol. However, the prevalence of BCG infection is not known, mainly because most laboratories cannot quickly differentiate between BCG and other members of the* M. tuberculosis *complex. Utilization of an allele-specific PCR combined with a multiplex PCR was found to be a sensitive and rapid test for the detection of* M. bovis *BCG in clinical specimens [37].

## 6. Complications of BCG Vaccination

BCG vaccine has been given to more than a billion people, but the protective efficacy is reported to vary in various human trials and the utility is further limited by their propensity to induce tuberculin reactivity [55, 56]. The current global threat of tuberculosis and the emergence of drug-resistant strains are compelling the scientific community to improve BCG vaccine or develop an entirely new vaccine against tuberculosis [57]. BCG vaccine has been considered to be safe, and although complications are rare after vaccination and the outcome is usually favourable, serious BCG infections can occur. Localized abscesses, regional lymphadenopathy, and disseminated disease in immunocompromised hosts are uncommon but well-recognized complications [58]. The retrospective review identified 60 cases of dissemination for which the mortality rate was 50%. BCG vaccine has been administered per cutaneous in Brazil since 1968 using the multiple puncture method. More than 1,000 publications made between 1921 and 1982 reported approximately 10,000 complications of BCG vaccination [58]. Recent molecular work has demonstrated differences between BCG and* M. tuberculosis *as well as within the BCG strains [59, 60]. Since BCG strains vary in protein expression [61], lipid composition [62], pathobiology in laboratory animals [63, 64] and humans, an understanding of genetic differences may provide insights into the determinants of protective immunity and vaccine associated complications [65–67].

The mild adverse reaction is characterized by a papule at an injection site, which may progress to become ulcerated. This may heal after 2–5 months leaving a superficial scar, and swelling of the epilateral regional lymph nodes may also occur. Multiple cutaneous lesions may signal disseminated BCG disease usually in an immunocompromised host [24]. Severe adverse events include subcutaneous abscess and keloids at the injection site and occurrence of a number of cutaneous lesions (such as TB chancre, lupus vulgaris, scrofuloderma, papulonecrotic, and disseminated tuberculosis) at the sites distinct from the vaccination site [68]. The incidence of local complications depends on the age of the recipient and the dose of vaccine. In newborn, BCG administration as an intradermal injection at any age is not easy; the commonest error is to inject the vaccine too deep. This deep injection can cause injection abscesses (2% cases). In more serious injection related complications, deep ulcers, osteomyelitis (0.04%), and lymphadenopathy (1%), especially in younger infants under one year, may occur. The immune dysfunction is directly related to disseminated disease, in the order of 1/1,000,000 doses, but is thought to be rare [69].

## 7. BCG Complications in HIV Infected Hosts

Following* M. bovis* BCG vaccination, development of disseminated disease in immune-compromised individuals has been reported which can be fatal in several cases [70]. The significantly high risk of disseminated BCG (dBCG) disease is reported in HIV-positive infants, with rates approaching 1% in South Africa [71]. Immune reconstitution inflammatory syndrome (IRIS) has recently been identified as a BCG vaccine-related adverse event in immunocompromised individuals after antiretroviral therapy (ART) [72]. The cellular primary immunodeficiency predisposes to the condition [73, 74]. The place of BCG vaccination in TB control programs is being carefully assessed as of the considerable risk of human to human transmission in immune-compromised patients, particularly in TB nonendemic countries [75–77].

## 8. BCG Vaccine and Tuberculin Skin Test (TST)

BCG-induced tuberculin reactivity is identical with reactivity induced by* M. tuberculosis* infection and the increased degree has been found in BCG revaccination in school children. The influence of BCG vaccination in past has been reported on tuberculin skin test (TST) surveys used as an auxiliary tool to estimate latent or active tuberculosis [78, 79].

## 9. Current Understanding of BCG Vaccination

The production of* M. bovis* BCG from different strains and by different manufacturers resulted in variable quality of vaccine and viabilities per dose of vaccine, as discussed in the previous paragraphs [80, 81]. Therefore, the World Health Organization is contemplating the revision in vaccine production guidelines, scope, terminology, and requirement of BCG vaccines. To discuss issues regarding the standardization, characterization of live and attenuated BCG vaccines, and evaluation of these vaccines, a consultative meeting of regulators, BCG vaccine manufacturers, researchers, and program managers was organized in 2010. The development of live attenuated TB vaccines, new recombinant BCG, and the characterization of different BCG sub-strains were also reviewed using state-of-the-art technologies to revise and update the various important issues related to current recommendations focused on the scope, terminology, manufacturing issues, and the incorporation of new reference reagents and new quality control test [82]. Interestingly, recent studies have shown that the combination of priming with recombinant BCG such as Δ*ure*C* hly* ^+^ rBCG and boosting it with most efficacious subunit vaccine would provide more powerful intervention measure against tuberculosis [83, 84]. The results of a long-term controlled trial of a BCG vaccine provides supports to investigators aspiring to produce vaccine with similar or improved characteristics as trial of a BCG vaccine found to have good protective efficacy against TB that extended up to 60 years after vaccination except some cases of pulmonary and extrapulmonary TB [85, 86].

## 10. Guidelines on Administration of BCG Vaccine

Tuberculosis emerged as a major concern in the aftermath of World War II, and subsequently, the use of BCG was encouraged in many countries, particularly by UNICEF and by Scandinavian Red Cross Societies and then by the WHO. Major trials were set up by the British Medical Research Council (BMRC) and by the United States Public Health Service (USPHS) in the early 1950s. The procedure employed by the BMRC provided high efficacy against tuberculosis [86, 87]. In contrast, BCG used by the USPHS (Park or Tice strains given to tuberculin-negatives of various ages) provided very little protection [55]. Respective public health agencies reported that BCG was recommended as a routine for tuberculin-negative adolescents in the UK, whereas in USA, BCG was restricted to certain high-risk populations but was not recommended for routine use [88]. Following major policy changes in the field of infectious disease control and immunization programs, and the amendment of the Immunization Law in 2001 BCG vaccination campaign was introduced [83, 89] according to various schedules (e.g., at birth, school entry, or school leaving) in the majority of countries [82].

## 11. Molecular Biology of BCG

### 11.1. Genetic Evolution

BCG is a derivative of* M. bovis *after the loss of the region of deletion 1 (RD1) that encodes the ESX-1 secretion system [90]. During the first half of the 20th century BCG was maintained by serial passage throughout the world, as mentioned in the history section. Over the decades, multiple BCG daughter strains were produced which resulted in several regions of genomic deletions as well as regions of genomic duplication and other mutations [83–86]. A tremendous opportunity is provided by the complete genome sequence of* M. tuberculosis *for investigating molecular mechanisms of overlapping disease manifestations produced by* M. bovis *BCG and* M. tuberculosis* and it is now evident that both share 99.9% of their DNA. It also shows that the BCG strain retained at least some of its original virulence characters [91–93]. The attenuation of BCG due to the loss of RD1 region from* M. bovis* and reintroduction of RD1 into BCG increased virulence significantly. Because of complementation neither BCG Pasteur nor the least passaged strain, BCG Russia, with RD1 resulted in the restoration of virulence to levels characteristic of* M. tuberculosis *or* M. bovis*. The reported genetic studies weaken the theory that the RD3, RD4, RD5, RD7, and RD9 loci are responsible for virulence among the tubercle bacilli [90]. The immune suppressive capacity of BCG is perhaps the most apparent feature in-vivo [87–91].

Some of the* M. bovis *BCG isolates that are reported to be sensitive to ethambutol, streptomycin, and p-nitrobenzoic acid reacted positively to cycloserine, but they are found to be resistant to isoniazid, rifampicin, pyrazinamide, and thiophen-2-carbonic acid hydrazide. However, lately, the cloning of pyrazinamidase gene (*pncA*) shows a single point mutation in the gene which is unique to* M. bovis *[94–98]. Therefore, to differentiate* M. tuberculosis *and* M. bovis* polymorphism, this gene could be a good option for diagnosis methods.

The standard mycobacterial culture techniques currently used in clinical microbiology laboratories are capable of identifying mycobacteria to the level of the* M. tuberculosis *complex.

It has been reported that most of* M. bovis *strainscontainspacers 40 to 43, whereas they lacks spacer 39 [36]. In 1993, Hoffner studied a high degree of biochemical heterogeneity within strains of the* M. tuberculosis *complex isolated [98], when subtyped by DNA fingerprinting using the insertion element IS*6110 *and spoligotyping [92]. Variable-number tandem repeats (VNTRs) occur throughout the chromosome of* M. tuberculosis*. Mycobacterial interspersed repetitive units (MIRUs) are polymorphic VNTRs and also have proved to be useful tools in molecular epidemiology; their biological significance is less well understood. The copy number of VNTR 3690 varies among Indian clinical isolates of* M. tuberculosis *(one to twelve copies),* M. tuberculosis *H37Rv TMC102 (four copies),* M. tuberculosis *H37Ra (two to four copies), and* M. bovis* BCG (one copy) [99]. A detailed comparison among virulent* M. tuberculosis*,* M. bovis,* and* M. bovis* BCG based on published literature [14, 15, 17–23] and on our own work is summarized in Table 1.

## 12. Conclusion

*M. bovis *strains are more virulent for cattle, while classical* M. tuberculosis *strains are thought to be more virulent for humans. The benefit of BCG immunization against* M. tuberculosis *infection has been the subject of much controversy. It is of uncertain efficacy and is associated with significant safety concerns in untreated HIV-infected infants and in those on ART.

The diagnosis and management of BCG disease are complex, leading to under recognition and suboptimal care in resource-limited settings often due to misdiagnosis. Better safety and efficacy profiles under investigations are highly needed for the new BCG vaccines. Vaccination policy attempt to balance risk and benefit needs to be revived. Various biochemical tests currently being used are useful methods for identifying* M. bovis* BCG virulence pathology, especially niacin positivity, which differs in the results of these tests among BCG substrains. The differences observed in different parts of the world could be attributed to the long passages of the BCG strains that have been subcultured in different laboratories leading to the divergence of* M. bovis* BCG strains in due course of time.

## Figures and Tables

**Table 1:** Characterization and comparison of different conventional biochemical and molecular tests to identify different *Mycobacterium bovis * (BCG) and *Mycobacterium tuberculosis* species.

Tests strain	*M. bovis* BCG P3	*M. tuberculosis* H37Rv	*Remarks *
Nitrate reduction	Negative	Positive	¥
Niacin	Positive	Positive	
Catalase	Negative	Negative	
*hsp65*-PCR	Positive	Positive	
*esat6*-PCR	Negative	Positive	
*Mac*-PCR	Negative	Negative	
MPT64 antigen	Negative	Positive	
MPT63 antigen	Positive	Positive	
Binary spoligotyping	■■□■■■■■□■■■■■■□■■■■■■■■■■■■■■■■■■■■■■□□□□□	■■■■■■■■■■■■■■■■■■■□□■■■■■■■■■■■□□□□■■■■■■■	
Octal number	676773777777600	777777477760771	
Shared type	482	451	
Lineage/sublineages	Bovis1_BCG	H37Rv	

Guinea pig virulence	Non-virulent	Virulent	Ψ

	*M. bovis *BCG (Tokyo)	*M. tuberculosis *H37Ra	

Nitrate reduction	Positive	Positive	[14]
Niacin	Positive^**^	Positive	
Catalase	Negative	Positive^**^	

	*M. bovis BCG* (ATCC 19274)*, M. bovis BCG *(Biken)	*M. tuberculosis H37Rv *(TMC102), H37Rv (Biken), H37Ra (Biken)	

Niacin test	Weakly Positive	Pos	[15]

	BCG Copenhagen, BCG Glaxo, BCG Pasteur, BCG Tice	*M. tuberculosis *H37Rv, *M. tuberculosis* H37Ra	

MPB64	Negative	Positive	[15]
MPB70	Positive	Positive	
16SrRNA	Positive	Positive	

	BCG Tokyo, BCG Moreau, BCG Russia, BCG Sweden*. M. bovis *AN5	—	

MPB64	Positive	—	[16]
MPB70	Positive	—	
16SrRNA	Positive	—	

	*M. bovis *BCG (Glaxo, Pasteur, Tice)	*M. tuberculosis *(H37Rv)	

MPB64	Negative	Positive	[16]
IS6110 copy number	1	—	

	*M. bovis *BCG (Brazilian, Japanese, Russian, Swedish)		

MPB64	Positive		[16]
IS6110 copy number	2	—	

	*M. bovis BCG *	*M. tuberculosis (classical) *	

Nitrate reduction	Positive	Positive	[17]
Niacin	Negative	Positive	

	*M. bovis *BCG	*M. tuberculosis *(classical)	

Nitrate reduction	Negative	Positive	[18]
Niacin	Negative	Positive	
MPB70	Positive	Positive	
Mtp40	Negative	Positive^*^ (very occasionally)	

	*M. bovis *BCG	*M. tuberculosis *	

Nitrate reduction	Negative	Positive	[19]
Niacin	Negative	Positive	
MPB70	Positive	Negative	
Mtp40	Negative	Positive	
Spoligotyping spacer 39–43	Negative	Positive	

	*M. bovis *	*M. tuberculosis *	

Nitrate reduction	Negative	Positive	[20]
Niacin accumulation	Negative	Positive	
Presence of mtp40	Negative	Positive	
Spoligotype (characteristic features)	At least one of spacers 39–43 present	Spacers 39–43 absent	

	*M. bovis *BCG	*M. tuberculosis *(H37Rv)	

Binary spoligotyping	■■□□□■■■□■■■■■■□■■■■□■■■■■■■■■■■■■■■■■□□□□□	■■■■■■■■■■■■■■■■■■■□□■■■■■■■■■■■□□□□■■■■■■■	[21]
Octal number	616773677777600	777777477760771	
Shared type	663	451	
	BOVIS1	H37Rv	

	*M. bovis *	*M. tuberculosis *	

Spacers 33 to 36 (derived from BCG)	—	*M. tuberculosis *does not hybridize to the spacers	[22]
Spacers 39 to 43 (derived from *M. tuberculosis*)	*M. bovis *and BCG do not hybridize to the spacers	—	

	*M. bovis *	*M. tuberculosis *(H37Rv)	

Capilia TB-Neo	Positive	Positive	[23]
SD MPT64	Positive	Positive	
TbcID	Positive	Positive	

	*M. bovis *BCG Tokyo	*M. bovis* BCG Connaught	

Capilia TB-Neo	Positive	Negative	[23]

^*¥*^Our analysis on the Indian seed-lot; ^Ψ^personal communication with the Director of the BCG vaccine producing laboratory; ^*^very occasionally positive; ^**^Weak Positive.
